# Development and validation of the newly developed Preschool Theory of Mind Assessment (ToMA-P)

**DOI:** 10.3389/fpsyg.2024.1274204

**Published:** 2024-04-08

**Authors:** I-Ning Fu, Cheng-Te Chen, Kuan-Lin Chen, Meng-Ru Liu, Ching-Lin Hsieh

**Affiliations:** ^1^Child Developmental Assessment and Intervention Center, Taipei City Hospital, Taipei, Taiwan; ^2^School of Occupational Therapy, College of Medicine, National Taiwan University, Taipei, Taiwan; ^3^Department of Occupational Therapy, College of Medicine, National Cheng Kung University, Tainan, Taiwan; ^4^Department of Educational Psychology and Counseling, National Tsing Hua University, Hsinchu, Taiwan; ^5^Institute of Allied Health Sciences, College of Medicine, National Cheng Kung University, Tainan, Taiwan; ^6^Department of Physical Medicine and Rehabilitation, College of Medicine, National Cheng Kung University Hospital, National Cheng Kung University, Tainan, Taiwan; ^7^Department of Physical Medicine and Rehabilitation, National Taiwan University Hospital, Taipei, Taiwan; ^8^Department of Occupational Therapy, College of Medical and Health Science, Asia University, Taichung, Taiwan

**Keywords:** theory of mind, multidimensional construct, assessment, preschool children, item response theory

## Abstract

**Introduction:**

Theory of mind (ToM) refers to the ability to understand and attribute mental states to oneself and others. A ToM measure is warranted for preschool children to assess their ToM development from a multidimensional perspective (i.e., cognitive and affective dimensions). This study aimed to develop the Preschool Theory of Mind Assessment (ToMA-P) and to evaluate its construct validity and applicability.

**Methods:**

The ToMA-P was developed based on comprehensive literature review and revised with expert panel feedback. Its psychometric properties were evaluated in 205 typically developing preschoolers with Rasch analysis for its dimensionality, item difficulties, and convergent validity.

**Results:**

The results indicated that all ToMA-P items, except for one, fit the hypothesized two-dimensional construct. The item difficulties in the cognitive and affective dimensions followed developmental sequences. The ToMA-P scores exhibited good convergent validity, as evidenced by its significant correlations with age, verbal comprehension, adaptive functions, and daily ToM performance (*p* < 0.05). Children’s responses and behaviors also showed that the ToMA-P has good applicability.

**Discussion:**

This study provides empirical evidence that the ToMA-P measures cognitive and affective ToM following developmental sequences, and that it has potential as a clinical tool for assessing ToM in preschool children.

## 1 Introduction

Theory of mind (ToM) is an essential ability that enables individuals to understand the mental states of others, such as beliefs, desires, and emotions. Individuals then use this information to recognize, forecast, and interpret the behaviors of others to navigate social situations successfully (Henry et al., 2013; Quesque and Rossetti, 2020). ToM serves as a cornerstone of social cognition (Mukerji et al., 2019), as it encompasses the capacity to perceive, identify, and interpret social cues guiding social behavior (Baillargeon et al., 2014; Lewis and Carpendale, 2014; Bowman et al., 2017). In the realm of cognitive social cognition, ToM plays a pivotal role in recognizing and interpreting social cues (Mcdonald, 2013). Frith and Frith (2008) further delineated ToM into implicit and explicit processes, those occurring with and without awareness, respectively. Individuals with ToM deficits may struggle with interpreting social cues and with developing and maintaining relationships, and these difficulties affect their social–emotional functioning, peer relationships, and school achievement (Lecce et al., 2011; Vissers and Koolen, 2016). Therefore, assessing children’s ToM development and identifying their ToM deficits is crucial for subsequent interventions (Wong, 2013; Beaudoin et al., 2020).

The ToM construct has been proposed to be both developmental and multidimensional based on neuroscientific evidence (Dvash and Shamay-Tsoory, 2014; Fu et al., 2023; Van den Stock et al., 2023). Children acquire ToM along a predictable path from birth to preschool years. During this period, children rapidly acquire developmental components of ToM, such as emotion distinction, diverse desires, diverse beliefs, first-order false belief, and second-order false belief (Wellman and Liu, 2004). These developmental components emerge at different ages. Children can recognize basic emotions by the age of 2 and rapidly develop ToM from the age of 3 years (Pons et al., 2004). Children learn to associate emotion words with facial expressions, known as the developmental component “emotion distinction” (Assed et al., 2020). Children start to understand that others may have desires and beliefs, and related emotions, divergent from their own, known as the developmental component “diverse desires/beliefs” (Wellman and Liu, 2004). At ages 4–6, children comprehend that others may have beliefs opposite to their own, which in turn lead to others taking contrary corresponding actions and having associated emotions, known as the developmental component “first-order false belief.” At 6–8 years of age, children are able to surmise that one person’s thoughts or emotions are opposite to those of another person, known as the developmental component “second-order false belief” (Astington et al., 2002). Understanding the developmental trajectory of ToM is important for generating a child’s ToM profile.

Theory of mind was initially viewed as a single construct and evaluated primarily using false belief tasks (Figueras-Costa and Harris, 2001). In recent years, ToM has been recognized as a multidimensional construct consisting of both cognitive and affective components (Westby and Robinson, 2014; Gabriel et al., 2021; Raimo et al., 2022; Zhou et al., 2022; Fu et al., 2023). Cognitive ToM refers to inferring others’ thoughts (e.g., What does Peter think is in the box?), while affective ToM means speculating on others’ emotions (e.g., How does Peter feel after he looks inside the box?) (Wellman and Liu, 2004; Altschuler et al., 2018; Baldimtsi et al., 2021). Cognitive ToM is a pre-requisite for affective ToM (Shamay-Tsoory et al., 2010). For example, in understanding “diverse desires,” a child has to infer two mental states: the individual’s preference for a specific food different from the child’s favorite, and the individual’s resulting happiness upon obtaining their preferred food. Since emotional inferences are often based on cognitive understanding of others’ behaviors or thoughts, children typically find inferring emotions more challenging than inferring thoughts within the same ToM developmental component task. Cognitive and affective ToM correspondingly overlap with cognitive and affective processes of social cognition. Cognitive social cognition covers inferring other’s beliefs and the intentions of others, while affective social cognition entails emotion recognition, emotion perception, and emotional empathy (Adolphs, 2010; Mcdonald, 2013). Different brain regions are, respectively, associated with cognitive and affective ToM. Cognitive ToM is attributed to several brain regions, namely the medial prefrontal cortex, superior temporal sulcus, temporoparietal junction, and temporal poles (Dvash and Shamay-Tsoory, 2014). Children with hearing-impairment and those with autism have been found to have impaired cognitive ToM, which affects their peer interactions by hindering their ability to understand others’ beliefs and behaviors (Peterson et al., 2012; Ziv et al., 2013). On the other hand, affective ToM is associated with other brain regions than cognitive ToM: the ventromedial and orbitofrontal cortices, ventral anterior cingulate cortex, amygdala, and ventral striatum (Abu-Akel and Shamay-Tsoory, 2011). Some clinical groups have affective ToM deficits, including children with oppositional defiance disorder, fetal alcohol syndrome, and autism spectrum disorders (de la Osa et al., 2016; Lindinger et al., 2016; Baldimtsi et al., 2021). These children struggle with identifying and interpreting emotional cues in facial expressions due to their affective ToM deficits (Hughes et al., 1998; Baribeau et al., 2015). For preschool children’s ToM profiles, researchers suggest that they be explored using both developmental and multidimensional ToM constructs (Baron-Cohen, 2000; Westby and Robinson, 2014; Fu et al., 2023).

Previous studies have shown that the ToM developmental components are subordinate to the ToM dimensions (Shamay-Tsoory et al., 2002; de la Osa et al., 2016; Fu et al., 2023). The subordinate relationship between ToM dimensions and developmental components is demonstrated by impaired performance on affective ToM tasks, but not on cognitive ToM tasks, in two different ToM developmental component tasks in clinical groups (Shamay-Tsoory et al., 2002; de la Osa et al., 2016). Additionally, cognitive ToM is a prerequisite for affective ToM within the same ToM developmental component. This concept is implied by people’s inference of emotions based on others’ possible behaviors or thoughts (Shamay-Tsoory et al., 2010). The aforementioned evidence indicates that both multidimensional and developmental ToM constructs should be considered when measuring preschool children’s ToM.

Various measures are available to evaluate ToM in preschool children, including compound measures such as the Strange Stories (Happé, 1994), ToM Scale (Wellman and Liu, 2004), Theory of Mind Task Battery (Hutchins et al., 2008a,b), Theory of Mind Booklet Task (Richardson et al., 2018), and ToM assessment scale in children (ToMas-child) (Rivas-Garcia et al., 2020). However, a recent systematic review suggests that these compound ToM measures lack particular items on the developmental components, respectively, in the cognitive and affective dimensions (Fu et al., 2023). For example, in the ToM Scale, the developmental component task “Diverse beliefs” assesses only cognitive ToM. From the developmental and multidimensional perspectives, these extant ToM measures cannot assess ToM developmental components concurrently in the cognitive and affective dimensions.

The presentation and response modes of the ToM measures define the requirements on children’s verbal comprehension ability and verbal expression ability. ToM measures that employ pictures, films, and cartoons may be more accessible to children with lower verbal comprehension abilities, while scenarios and spoken stories may be more challenging (Adachi et al., 2004; Harper-Hill et al., 2014; Shahaeian et al., 2014; Fu et al., 2023) Multiple-choice questions with pictures or objects may also be easier for children with lower verbal expression abilities, while responses requiring spoken language may be more difficult (Pons et al., 2004; Wellman and Liu, 2004; Sivaratnam et al., 2012; Richardson et al., 2018; Fu et al., 2023). To support children with poor verbal abilities, visual aids are recommended for use in ToM measures to help children understand the stories better and answer the questions more easily (Fu et al., 2023).

Classical test theory (CTT) and item response theory (IRT) are commonly used in developing ToM measures. Most existing ToM measures were developed using CTT and take the total item score as the scale score, providing test-level information (Happé, 1994; Pons et al., 2004; Sabbagh et al., 2009; Bowman et al., 2017; Borbás et al., 2021; Fu et al., 2023). However, treating ordinal scores as interval variables may lead to inappropriate analytic methods, doubtful interpretations of inappropriate analytic methods, and doubtful interpretations of measurement results (Shih et al., 2013). To overcome these limitations, IRT, particularly Rasch modeling, has been developed (Rasch, 1960; Shih et al., 2013). Rasch analysis provides both item-level information and test-level information. The original ordinal scores can be transformed into an interval scale, precisely reflecting differences between a participant’s scores, repeated assessments, and changes over time (Shih et al., 2013). Consequently, the Rasch model is considered appropriate for constructing interval scales and evaluating the psychometric properties of a measure. However, only a few ToM measures have been constructed based on the Rasch model (Fu et al., 2023).

To conclude, existing ToM measures for preschool children should address three issues. First, the ToM construct should be both multidimensional and developmental. Second, visual aids are suggested for utilization in the presentation and response modes for children with poor verbal abilities. Finally, IRT is recommended as the appropriate psychometric methodology for constructing a ToM measure with interval scales. Thus, a new ToM measure for preschool children is warranted to overcome the limitations of existing measures. This new measure should classify ToM difficulties according to cognitive and affective dimensions while incorporating developmental components, employing visual aids in the presentation and response modes, and utilizing IRT. This study aimed to develop the Preschool Theory of Mind Assessment (ToMA-P), and to evaluate the construct validity and applicability of the ToMA-P.

## 2 Methods

### 2.1 Developing the Preschool Theory of Mind Assessment (ToMA-P)

#### 2.1.1 Item design

Drawing upon research into the developmental trajectory of ToM, the ToMA-P comprises four core ToM developmental components tailored for preschool children: emotion distinction, diverse desires, unexpected location, and second-order false belief (Baron-Cohen, 2000; Wellman and Liu, 2004; Blijd-Hoogewys et al., 2008; Hutchins et al., 2008a,b). The ToMA-P has eight items in two dimensions of cognitive ToM (three items) and affective ToM (five items) to measure four core developmental components that develop in the preschool years, including emotion distinction (two affective ToM items), diverse desires (one cognitive ToM item; one affective ToM item), unexpected location (one cognitive ToM item; one affective ToM item), and second-order false belief (one cognitive ToM item; one affective ToM item). Each item belongs to one ToM dimension and one developmental component. The numbers of items in each dimension with developmental components vary due to the definition of each developmental component. For example, the developmental component “emotion distinction” involves children’s ability to differentiate and label facial expressions of emotions, which does not align with the definition of cognitive ToM (thinking about the thoughts of others). Therefore, no items of the developmental component “emotion distinction” can be designed for the cognitive ToM dimension.

The ToMA-P was developed using the most widely used representative ToM tasks in previous studies, with consideration of children’s attention spans. The items belonging to emotion distinction, diverse desires, unexpected location, and second-order false belief were, respectively, based on the control emotion items of the Comic Strip Task, ToM Scale, Sally-Anne task, and ice-cream van task (Perner and Wimmer, 1985; Wellman and Liu, 2004; Sivaratnam et al., 2012). These items were further redesigned based on multidimensional constructs and Taiwanese culture (Dvash and Shamay-Tsoory, 2014; Westby and Robinson, 2014). The items in the ToMA-P are designed with objects and events familiar to Taiwanese children to avoid cultural differences. This consideration of cultural issues encompasses three categories: foods, character names, and leisure activities. For example, previous ToM stories often featured foods like hamburgers and hot dogs, which are common in Western society. In contrast, the ToMA-P includes Eastern foods such as dumplings, pudding, and Taiwanese sausage. In addition, the character names in previous ToM stories were typically common English names such as John, Mary, and Linda; however, the ToMA-P uses names like Da-Ming (大明) and Mei-Mei (美美), which are more familiar to Taiwanese children. Additionally, leisure activities preferred by Taiwanese children, such as playing games on handheld consoles, were incorporated into the ToMA-P. As it considers these cultural differences, the ToMA-P is tailored to the experiences and preferences of Taiwanese children, ensuring its applicability in this context.

**Figure 1:**
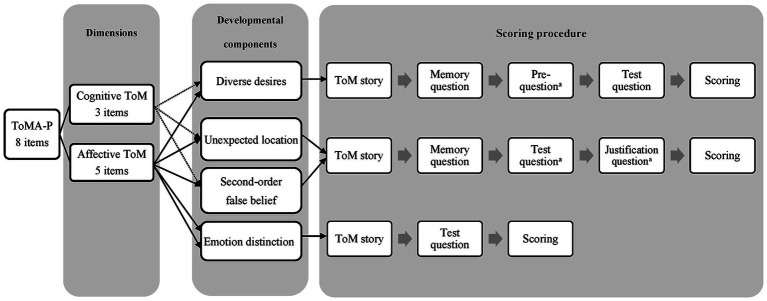
Measurement structure and scoring procedure of the Preschool Theory of Mind Assessment. Questions can be provided to the children only when the previous questions are answered correctly.

**Figure 2:**
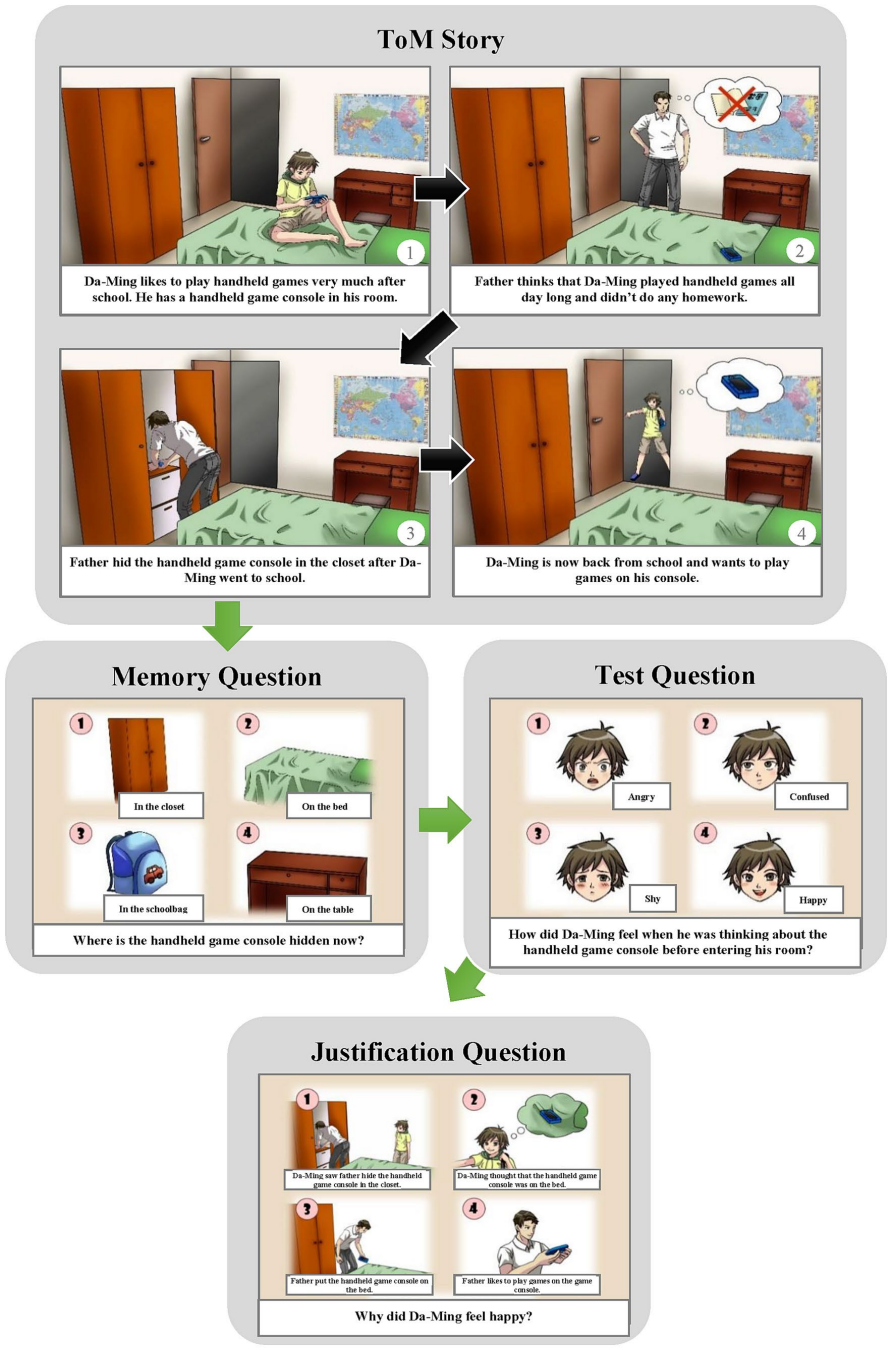
Example of the item “Affective theory of mind, Unexpected location (A–U).” The original items are presented in Mandarin Chinese.

Each item is presented using full-color comic strips that illustrate daily social situations related to the family of Da-Ming, the central character. The child being assessed has to infer the mental states, behaviors, or emotions of the characters. The comic strips are interactive and presented on a computer for a more engaging experience and are more efficient than traditional booklets. After each story, 2–4 multiple-choice questions are presented in pictures, including memory, test, justification, and pre-questions. Memory questions, which are about reality or expression or someone else’s state, are used to confirm that the children remember the story that was told. Test questions, which are about the protagonist’s mental state or behavior or emotion, are used as a quicker way of testing ToM. Justification questions, which are about the reasons for the protagonist’s mental state or behavior, are used to better reveal the ToM information of children. Pre-questions, which are about the material or belief that the children prefer and are followed by a test question, are used to complete the story. The multiple-choice answering mode enables children with poor verbal abilities to point to the answer, revealing their true ToM. Children who pass all the questions in each item can get 1 point; otherwise, they get a score of 0. The total score range of the ToMA-P is 0–8. Figure 1 shows the measurement structure and scoring procedure of the ToMA-P. Table 1 shows the scoring details of each set of questions. Figure 2 presents an example of the “Affective ToM, Unexpected location” item.

**Table 1:** Questions and scoring of the Preschool Theory of Mind Assessment.

Component	Set of questions	Scoring
Diverse desires	Memory question, pre-question, test question.	1: Correctly answer memory question and test question
	Pre-question and test question can be provided to the children only when the memory questions are answered correctly.^a^	0: Other conditions
Emotion distinction	Test question.	1: Correctly answer test question
		0: Other condition
Unexpected location	Memory question, test question, and justification question.	
Second-order false belief	Test questions can be provided to the children only when the memory questions are answered correctly. ^a^	1: Correctly answer memory question, test question and justification question
	Justification questions can be provided to the children only when the test questions are answered correctly.^a^	0: Other conditions

^a^represents the order of the questions.

#### 2.1.2 Item construction and revision

After identifying the most widely used representative ToM tasks and multidimensional constructs, the first author and second author developed the initial items based on two levels: (1) the overall assessment level: the measurement structure and scoring rules; (2) the item level: the item format, the corresponding developmental components and multidimensional dimensions, the feasibility of the item format, the appropriateness of the items for preschool children, and sentence fluency. Then four experts familiar with the construct and child development of ToM evaluated the items according to the same two-level principles. The items of the ToMA-P were subsequently revised through back-and-forth discussion until all their comments were addressed. Finally, the written format of the ToMA-P was finalized for transformation into comic strips by a senior pediatric occupational therapist with 10 years of comic drawing experience.

### 2.2 Participants

Typically developing (TD) Taiwanese children aged 3–6 years were invited to participate in this study. To be included, children had to be considered TD by their caregivers and teachers. Exclusion criteria were (1) neurodevelopmental disease, such as autism spectrum disorders, (2) Verbal Comprehension Index scores on the Wechsler Preschool and Primary Scale of Intelligence-IV of below 85 (percentile rank <15, Mean − 1 SD), (3) children with uncorrectable visual or auditory impairment, and (4) unfamiliarity with Mandarin Chinese.

### 2.3 Measures

#### 2.3.1 Preschool Theory of Mind Assessment (ToMA-P)

The ToMA-P completed in the revision step was used in Phase 2.

#### 2.3.2 Verbal Comprehension Index (VCI) of the Wechsler Preschool and Primary Scale of Intelligence-IV (WPPSI-IV)

Children’s verbal IQs were calculated from the VCI of the WPPSI-IV. The VCI was designed to measure the children’s verbal knowledge and verbal reasoning ability. In the current study, the VCI was used to exclude children who had poor verbal comprehension ability and to examine the convergent validity of the ToMA-P. The half-split reliability, test–retest reliability, internal consistency, inter-rater reliability, and convergent validity of the WPPSI-IV are well examined in Taiwanese populations (Chen et al., 2007).

#### 2.3.3 Applicability Questionnaire (AQ)

The AQ was used to gather the children’s perceptions and feelings regarding the ToMA-P for examination of its applicability. The AQ contained questions about the ToMA-P to gather information on the following: (1) the likeability of the plots, characters, and objects in the stories; (2) the appropriateness of the words and sentences, and options that the children could not understand or recognize; and (3) the answering burden. In addition, the number and durations of breaks requested by the children and the number and durations of distraction (children’s line of sight straying from the computer screen for over 5 s) were also recorded in the AQ. The children were interviewed with the AQ after the assessment of the ToMA-P. The responses of the children were used to examine the applicability of the ToMA-P.

#### 2.3.4 Vineland Adaptive Behavior Scale (VABS)

The VABS was used to evaluate the adaptive function of the children. The VABS contains four main domains: communication, daily living skills, socialization, and motor skills. The standard scores of the communication and socialization subscales were employed to examine the convergent validity. The Chinese version of the VABS has been reported to have great split-half reliability (0.91–0.99) and good test–retest reliability (0.74–0.89) (Wu et al., 2004).

#### 2.3.5 Chinese version of the Theory of Mind Inventory-2 (ToMI-2-C)

The ToMI-2-C was used to measure the children’s daily ToM performance in real social contexts. The ToMI-2-C is designed for children aged from 3 to 12 years and completed by caregivers. The 60 items are distributed in three subscales that follow the development of ToM stages: early, basic and advanced. The basic subscale score of the ToMI-2-C was employed for examination of the convergent validity due to the target age group in the present study. The ToMI-2-C is a comprehensive daily ToM performance measure that can reflect developmental progression, and it also has high internal consistency (Cronbach’s α = 0.96), appropriate test–retest reliability (ICC = 0.88), moderate convergent validity (0.43–0.59), and good discriminative validity for differentiating children with autism spectrum disorders and those with typical development (Lee et al., 2021, 2023; Chen et al., 2023).

### 2.4 Procedures

The study was approved by the Institutional Review Board of the National Cheng Kung University Hospital (B-BR-105-020-T). The children were enrolled in this study if their legal guardians gave consent. The children were assessed individually in separate classrooms within their kindergartens, first with the VCI of the WPPSI-IV and then with the ToMA-P. The VCI of the WPPSI-IV was provided and recorded by the administrator. Then, the ToMA-P was presented on the computer screen and recorded by the administrator. The VCI of the WPPSI-IV and the ToMA-P took about 40 min, including a short break. After the ToMA-P assessment, each child was asked the questions about the applicability. The children’s primary caregivers were asked to fill out a demographics form, the VABS, and the ToMI-2-C.

### 2.5 Data analysis

A two-factor model of the between-item multidimensional random coefficients multinomial logit model (MRCMLM) was constructed to examine whether the ToMA-P measures two-dimensional ToM. The between-item MRCMLM states a correlation structure between domains in its model formulation to deliver multiple responses from the same participant. ConQuest and SPSS computer software were used for data analysis. When the data fit the model’s expectation, the infit (weighted) and outfit (unweighted) mean square error (MnSq) have an expected value of unity. MnSq statistics between 0.7 and 1.3 were considered to indicate a reasonably good model–data fit (Wright and Linacre, 1994). Items with infit or outfit MnSq statistics beyond this range are usually regarded as misfitting.

The relation between item difficulty and person ability was tested by the mean participant ability of individual aspects, regarding the total difficulty in each aspect, and by floor and ceiling effects. A floor effect was considered more than 20% of the participants scoring 0 on all items, while a ceiling effect was defined as more than 20% scoring 1 on all items.

Regarding the convergent validity, the factors of age, verbal comprehension ability, daily ToM performance, verbal communication, and social functioning were assumed to be positively associated with ToM, based on previous evidence (Wellman and Liu, 2004; Blijd-Hoogewys et al., 2008; Peterson et al., 2012; Bishop-Fitzpatrick et al., 2017). For the examination of convergent validity, multidimensional latent regression was used to examine the relations in the means of the ToMA-P scores with age, VCI scores, basic subscale scores of the ToMI-2-C, the standard scores of the communication, and the socialization subscales of the VABS. A predictor with a *p*-value <0.05 was deemed meaningful to the model.

Differential item functioning (DIF) refers to the phenomenon that children from different groups of equal ability may have different probabilities of passing an item (Chen and Revicki, 2014). The DIF-free-then-DIF strategy was used to select a set of items (or an item) that were (was) the most likely to be DIF-free, and the other items were assessed for DIF using the designated item(s) as anchor(s). Once a difference was found between gender groups, the item was considered as exhibiting DIF. *Z*-test was used to examine the estimates between gender groups, and a low *p* value (< 0.05) was deemed a sign of substantial DIF (Shih et al., 2013). Misfitting items exhibiting DIF were revised or omitted from the ToMA-P based on the results of fit statistics, an item–person map, and DIF analysis.

Regarding the applicability, the data of the AQ were synthesized using a narrative and quantitative format, which recorded the level of likeability, appropriateness, and burden of answering. The number of breaks requested by the children and the durations of distraction during administration were also recorded, and fewer than three times was deemed acceptable for preschool children.

## 3 Results

### 3.1 Descriptive statistics

A total of 215 children aged from 3 to 6 years were recruited from nine preschools in Tainan, Taiwan. Ten children were excluded because their VCI scores were below 85. A total of 205 TD children (mean age = 58.91 months, SD = 11.49 months, range = 37–79 months), comprising 101 boys, were included for analysis. Table 2 demonstrates the gender and age distribution of the typically developing sample. A subgroup of 141 children completed the VABS and ToMI-2-C for examination of the convergent validity. In all, 51% of the children’s fathers were college graduates, and 69.9% of the children’s mothers were college graduates. Most family incomes were between NTD 50,000 and 100,000 per month, which is the average level of a family’s monthly income in Taiwan (Directorate-General of Budget, Accounting and Statistics, Executive Yuan, Republic of China, 2021). Table 3 demonstrates the descriptive results of the measures used in the present study. Table 4 shows the total pass rates of each item in the ToMA-P.

**Table 2:** Gender and age distribution of the typically developing sample (*n* = 205).

Age group	Boys/girls: n (%)	Mean Age: months (SD)
3 years 0 months–3 years 5 months	5 (31.3%)/11 (68.8%)	39.19 (1.79)
3 years 6 months–3 years 11 months	14 (58.3%)/10 (41.7%)	44.17 (1.78)
4 years 0 months–4 years 5 months	14 (48.3%)/15 (51.7%)	50.21 (1.78)
4 years 6 months–4 years 11 months	20 (52.6%)/18 (47.4%)	57.21 (1.75)
5 years 0 months–5 years 5 months	15 (48.4%)/16 (51.6%)	62.42 (1.74)
5 years 6 months–5 years 11 months	15 (48.4%)/16 (51.6%)	68.61 (1.90)
6 years 0 months–6 years 7 months	18 (50.0%)/18 (50.0%)	74.94 (1.98)

**Table 3:** Descriptive data of all participants in the present study (*n* = 205).

Variables	Mean (SD, range)
ToMA-P	
Cognitive dimension score	1.12 (0.74, 0–3)
Affective dimension score	2.75 (0.82, 0–5)
Total score	3.86 (1.34, 0–8)
WPPSI-IV-VCI	111.20 (15.07, 86–151)
ToMI-2-C-Basic subscale score (*n* = 141)	15.61 (2.38, 7.30–20.00)
VABS (*n* = 141)	
Raw score	
Communication subscale	74.34 (15.10, 31–120)
Socialization subscale	61.88 (17.68, 28–105)
Standard score	
Communication subscale	107.41 (19.67, 69–145)
Socialization subscale	105.33 (22.72, 61–145)

ToMA-P, Preschool theory of mind assessment; WPPSI-IV-VCI, Verbal Comprehension Index scores of the Wechsler Preschool and Primary Scale of Intelligence-IV; ToMI-2-C, Chinese version of the Theory of Mind Inventory-2; VABS, Vineland Adaptive Behavior Scale.

**Table 4:** Total pass rate of items in the Preschool Theory of Mind Assessment (*n* = 205).

Items	Pass rate: n (%)
Cognitive dimension	
Cognitive ToM, Diverse desires (C-D)	164 (76.2%)
Cognitive ToM, Unexpected location (C-U)	43 (20.0%)
Cognitive ToM, Second-order false belief (C-S)	28 (13.0%)
Affective dimension	
Affective ToM, Emotion distinction: Happy (A-E1)	206 (95.8%)
Affective ToM, Emotion distinction: Sad (A-E2)	202 (93.9%)
Affective ToM, Diverse desires (A-D)	134 (62.3%)
Affective ToM, Unexpected location (A-U)	38 (17.7%)
Affective ToM, Second-order false belief (A-S)	5 (2.3%)

### 3.2 Construct validity

#### 3.2.1 Model–data fit

Table 5 shows the fit statistics of the ToMA-P items. All but the item “Affective ToM, Second-order false belief (A-S)” (outfit MnSq = 2.34) were found to fit the model with the accepted infit statistics of 0.91–1.11 and outfit statistics of 0.86–1.24.

**Table 5:** Fit statistics of the Preschool Theory of Mind Assessment.

	Estimate	Standard error	Infit statistics		Outfit statistics	
			MnSq	Z std	MnSq	Z std
Cognitive dimension						
C-D	−2.48	0.14	1.02	0.2	1.20	1.9
C-U	0.96	0.14	1.00	−0.0	0.98	−0.2
C-S	1.52	0.20	1.02	0.2	0.87	−1.4
Affective dimension						
A-E1	−3.71	0.29	1.01	0.1	0.97	−0.3
A-E2	−3.32	0.27	1.02	0.1	1.24	2.3
A-D	−0.34	0.16	0.91	−1.3	0.86	−1.5
A-U	2.39	0.18	1.04	0.4	1.23	2.2
A-S	4.96	0.45	1.11	0.4	2.34*	10.0

C-D, Cognitive ToM, Diverse desires; C-U, Cognitive ToM, Unexpected location; C-S, Cognitive ToM, Second-order false belief; A-E1, Affective ToM, Emotion distinction: Happy; A-E2, Affective ToM, Emotion distinction: Sad; A-D, Affective ToM, Diverse desires; A-U, Affective ToM, Unexpected location; A-S, Affective ToM, Second-order false belief; Estimate, Item difficulty; MnSq, Mean square error; ^*^indicates misfitting item (MnSq > 1.3 or MnSq < 0.7).

#### 3.2.2 Item difficulty

**Figure 3:**
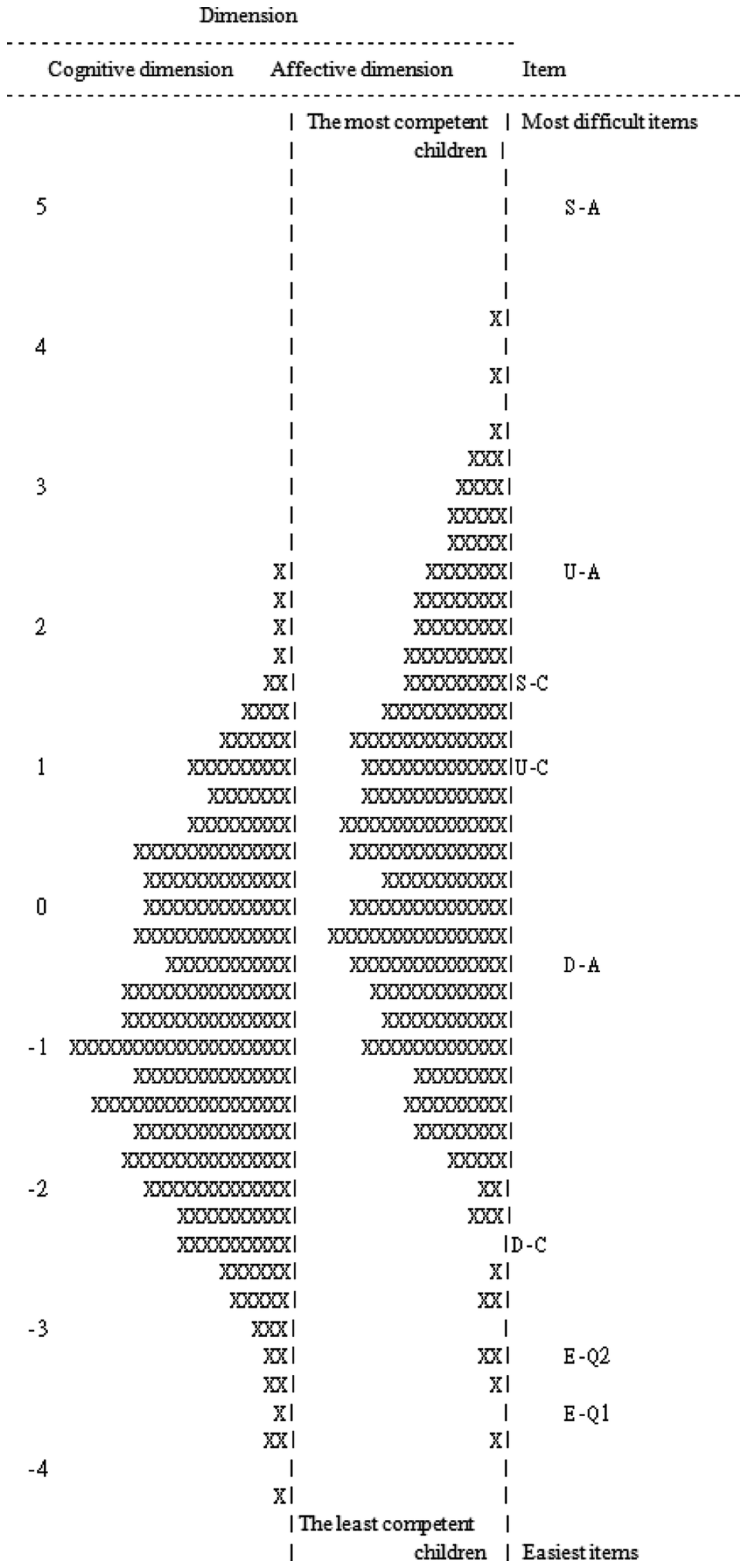
Item-person map. Each “X” represents 0.8 cases.

Figure 3 illustrates the item–person map to describe the relation between item difficulty and person ability. The items of the cognitive dimension are listed in descending order of difficulty as follows: Cognitive ToM, Second-order false belief (C-S); Cognitive ToM, Unexpected location (C-U); and Cognitive ToM, Diverse desires (C-D). On the other hand, the item difficulties of the affective dimension, in descending order, are as follows: A-S; Affective ToM, Unexpected location (A-U); Affective ToM, Diverse desires (A-D); Affective ToM, Emotion distinction: Sad (A-E2); and Affective ToM, Emotion distinction: Happy (A-E1). The most difficult items in the cognitive and affective dimensions are, respectively, item C-S and item A-S; the easiest items are, respectively, item C-D and item A-E1. In addition, no floor and ceiling effects were found in the ToMA-P. Only 1.4% of the children’s scores on all items were 0, while 0.5% of the participants scored 1 on all items.

#### 3.2.3 Differential item functioning

The items C-U and A-E1 were, respectively, chosen as the DIF-free anchors in the cognitive and affective dimensions, and none of the items in the ToMA-P were found to exhibit DIF (*p* > 0.05). Item functioning did not differ between genders.

#### 3.2.4 Convergent validity

With regard to the convergent validity, we found that age, VCI score, the basic subscale score of the ToMI-2-C, and the standard scores of the communication and socialization subscales of the VABS positively significantly predicted the cognitive dimension scores of the ToMA-P (regression coefficients = 0.082, 0.048, 0.237, 0.022, 0.020, all *p* < 0.001), and the affective dimension scores of the ToMA-P (regression coefficients = 0.086, 0.056, 0.239, 0.025, 0.021, all *p* < 0.001).

### 3.3 Applicability

Regarding the likeability of the ToMA-P, 92% of the children mentioned that they liked the plots, characters, or objects in the stories. With regard to the appropriateness of the words, sentences, and options, 69.5% of the children reported that they understood all of the words and sentences in the ToMA-P, and 71.8% of the children stated that they could choose a correct answer from the options. Regarding the burden of answering of the ToMA-P, 77% of the children expressed that they did not feel a burden after completing the ToMA-P. Regarding the number and durations of breaks requested by the children and the durations of distraction during administration, fewer than three times was deemed acceptable due to the concentration ability of preschool children. Most children (80.8%) did not request any breaks during the administration, and only one child requested three breaks to listen to the stories in the ToMA-P again. On the other hand, 76.5% of the children were not distracted during the administration, and 5.1% of the children were distracted more than three times during the administration. With regard to the children who were distracted more than three times during the administration, 36% were aged between 3 years 0 months and 3 years 11 months, 45% were aged between 4 years 0 months and 4 years 11 months, and 19% were aged between 5 years 0 months and 6 years 7 months. Children below 5 years old accounted for the largest proportion of the children who were distracted and exceeded the acceptable level during the administration.

## 4 Discussion

The present study presents the development and psychometric examination of a newly developed ToM measure for preschool children, the ToMA-P. The ToMA-P was developed with three advantages to overcome the issues of the previous ToM measures. First, the ToMA-P assesses ToM from both developmental and multidimensional perspectives. Second, the ToMA-P has visual aids and can be more appropriate for children with poor verbal comprehension or expression ability; therefore, it can reveal the true ToM of the children. Third, the ToMA-P was constructed using IRT, which is appropriate for constructing interval scales and provides both item-level information and test-level information. In addition, our findings demonstrate that the ToMA-P has good validity and applicability. The ToMA-P is a novel, engaging, and practical measure of ToM for preschool children from both developmental and multidimensional perspectives.

The good fit with the two-dimensional model represented the underlying two-dimensional ToM construct, which was in line with the known two-dimensional construct (Shamay-Tsoory et al., 2010; Westby and Robinson, 2014). In addition, the good model–data fit indicated that the ToMA-P can measure the two-dimensional ToM construct, thus indicating good construct validity. The only misfitting item, item A-S, fell within the accepted range for infit statistics, but it fell outside the accepted range for outfit statistics. The outfit statistic (outlier-sensitive fit statistic), which reports large differences between observed and expected values for items that are far beyond the person’s ability (Stelmack et al., 2004), is more sensitive to unexpected observation of items with difficulty beyond a person’s ability. A high outfit statistic may be the result of a few random responses by low or high performers, such as carelessness or lucky guessing by extreme performers. For item A-S, the low performers might have simply guessed and chosen the right answer by chance. This is supported by the fact that two of the five children who passed item A-S did not pass all of the items belonging to early ToM, leading to the high outfit statistic of item A-S. Outfit problems are less of a threat to measurement than infit problems are. The misfitting item A-S was retained to maintain the integrity of item difficulty for the ToMA-P.

The total pass rates of the items and item difficulty of the present study followed the developmental sequence and multidimensional construct of ToM. The developmental ToM components are listed according to the item difficulties in both dimensions in the present study as follows: emotion distinction, diverse desires, unexpected location, and second-order false belief. The developmental sequence reported above is the same as that in previous studies (Peterson et al., 2005; Wellman et al., 2011; Westby and Robinson, 2014). In addition, no floor or ceiling effects were found in the ToMA-P. From item difficulty evidence based on IRT, the ToMA-P also has the potential to assess developmental changes in ToM in longitudinal studies. In addition, the results of the total pass rate of the items indicated that, for the same ToM developmental component, the affective items are always more difficult than the corresponding cognitive items in the ToMA-P. The total pass rates of the cognitive and affective items of the present study were consistent with the concept that cognitive ToM is a prerequisite for affective ToM (Shamay-Tsoory et al., 2010). In addition, our results showed that ToMA-P scores were associated with age, verbal comprehension ability, daily ToM performance, verbal communication, and social functioning, which is consistent with previous research (Wellman and Liu, 2004; Blijd-Hoogewys et al., 2008; Peterson et al., 2012; Bishop-Fitzpatrick et al., 2017). Therefore, the ToMA-P revealed impressive construct validity.

The ToMA-P was found to be engaging and interactive for preschool children. The scenes, plots, character design, and character names were fascinating and reasonable, which promoted children’s engagement. For instance, in item C-D, children were asked which food they wanted to eat, and then A-Bao would choose a different food. This interactive element was exciting for children and kept them interested in subsequent plots. Unlike general comic strips or storybooks, children were called upon to interact with the ToMA-P stories actively, which made them more engaging. In addition, most children reported that they enjoyed and understood the stories without feeling burdened. The reasons for children’s enjoyment were the appearance of their favorite foods, characters, and objects, the interesting plots, and the beautiful pictures. The plots were interesting because they reflected the daily lives of Taiwanese children, and the relatability of the stories made it easier for children to engage with them. The reading speed of the ToMA-P was appropriate for most children, allowing them sufficient time to comprehend the stories and choose the right answer. However, some young preschoolers found the speed too fast, which could result in a burden. To address this, administrators should confirm the reading speed with young children and read the stories at a slower pace in subsequent administrations. The attractiveness of the ToMA-P minimized distractions during the administration, as most children were fascinated by the stories and tried their best to understand and answer them, which helped to measure their true ToM ability.

Although the ToMA-P is a practical ToM measure for assessing ToM abilities in preschool children, a few limitations should be considered in the interpretation of these findings. First, the sample size in the present study was insufficient, especially the inadequate number of children aged 3 years 0 months to 3 years 5 months. With larger and more representative samples, more robust item parameter estimates could be obtained using Rasch modeling to enhance the accuracy of the item difficulty estimation. Second, the children’s memory function was not evaluated and might be an element of uncertainty during the evaluation of ToM with the ToMA-P. Since basic memory function is required to remember the stories in the ToMA-P and then answer questions, the item difficulties in the ToMA-P may be increased by the demand on memory function. Finally, for the evaluation of the convergent validity of the ToMA-P, we applied a parent-report questionnaire, the ToMI-2-C, to assess the children’s daily ToM performance. The ToMI-2-C assesses the ToM construct from the parents’ perspective and has been found to be significantly correlated with the ToMA-P, which is directly administered to children (Lee et al., 2019). In future studies, additional standardized ToM measurements directly administered to the children can further enhance the robustness of the evidence of the validity of the ToMA-P.

Based on the preliminary evidence of the validity and applicability, the ToMA-P seems to be an attractive, applicable, and valid measure to evaluate comprehensively the ToM of preschool children, including both developmental and multidimensional perspectives, as both perspectives are critical to comprehensive understanding of children’s ToM. The ToMA-P possesses clear advantages over previous ToM tests. While a few existing ToM measures have adopted developmental and multidimensional constructs, they lack specific items addressing developmental components, respectively, in both cognitive and affective dimensions. Consequently, they cannot concurrently assess ToM developmental components in these two dimensions (Happé, 1994; Wellman and Liu, 2004; Hutchins et al., 2008a,b; Richardson et al., 2018; Rivas-Garcia et al., 2020). In contrast, the ToMA-P was meticulously designed with specific items targeting developmental components in both dimensions. With this measure, researchers will be able to explore how the dimensions and developmental components affect one another and further examine the mechanisms governing the dimensions and developmental components. In addition, clinicians will be able to understand children’s ToM in the two dimensions or developmental components and plan corresponding interventions in clinical populations, such as children with autism spectrum disorders and social communication disorders.

## Data availability statement

The original contributions presented in the study are included in the article/supplementary material, further inquiries can be directed to the corresponding author.

## Ethics statement

The studies involving humans were approved by Institutional Review Board of the National Cheng Kung University Hospital. The studies were conducted in accordance with the local legislation and institutional requirements. Written informed consent for participation in this study was provided by the participants’ legal guardians/next of kin.

## Author contributions

I-NF: Conceptualization, Funding acquisition, Methodology, Project administration, Writing – review & editing, Data curation, Formal Analysis, Investigation, Visualization, Writing – original draft. C-TC: Methodology, Supervision, Writing – review & editing, Formal Analysis, Validation. K-LC: Methodology, Supervision, Writing – review & editing, Conceptualization, Funding acquisition, Project administration, Resources. M-RL: Data curation, Writing – review & editing. C-LH: Methodology, Supervision, Validation, Writing – review & editing.
